# Anomalous Decay of Quantum Resistance Oscillations of 2D Helical Electrons in Magnetic Field

**DOI:** 10.1038/s41598-020-64385-7

**Published:** 2020-05-12

**Authors:** S. Abedi, S. A. Vitkalov, N. N. Mikhailov, Z. D. Kvon

**Affiliations:** 1 Physics Department, City College of the City University of New York, New York, 10031 USA; 2 A.V.Rzhanov Institute of Semiconductor Physics, Novosibirsk, 630090 Russia; 3 Novosibirsk State University, Novosibirsk, 630090 Russia

**Keywords:** Topological insulators, Two-dimensional materials

## Abstract

Shubnikov de Haas resistance oscillations of highly mobile two dimensional helical electrons propagating on a conducting surface of strained HgTe 3D topological insulator are studied in magnetic fields *B* tilted by angle *θ* from the normal to the conducting layer. Strong decrease of oscillation amplitude *A* is observed with the tilt: ${\boldsymbol{A}}\sim {\boldsymbol{e}}{\boldsymbol{x}}{\boldsymbol{p}}(\,-\,{\boldsymbol{\xi }}/{\boldsymbol{c}}{\boldsymbol{o}}{\boldsymbol{s}}({\boldsymbol{\theta }}))$, where *ξ* is a constant. Evolution of the oscillations with temperature *T* shows that the parameter ${\boldsymbol{\xi }}$ contains two terms: ${\boldsymbol{\xi }}={{\boldsymbol{\xi }}}_{1}+{{\boldsymbol{\xi }}}_{2}{\boldsymbol{T}}$. The temperature independent term, ${{\boldsymbol{\xi }}}_{{\bf{1}}}$, signals possible reduction of electron mean free path ${l}_{q}$ and/or enhancement of in-homogeneous broadening of the oscillations in magnetic field *B*. The temperature dependent term, ${{\boldsymbol{\xi }}}_{{\bf{2}}}{\boldsymbol{T}}$, indicates increase of the reciprocal velocity of 2D helical electrons: $\delta ({v}_{F}^{-1})\sim B$ suggesting modification of the electron spectrum in magnetic fields. Results are found in good agreement with proposed phenomenological model.

## Introduction

Two- and three-dimensional topological insulators (3D TIs) represent a new class of materials with an insulating bulk and topologically protected conducting boundary states^1–10^. In 3D TIs, due to a strong spin-orbit interaction, a propagating surface electron state with wave vector *k* is non-degenerate and keeps the electron spin polarization locked perpendicular to the wave vector *k* in the 2D plane (2D helical electrons)^5,9,10^. Due to the spin-momentum locking, the electron scattering on impurities is suppressed since the scattered electron should change both the linear and the angular (spin) momenta. It leads to a topological protection of the helical electrons against the scattering. In particular, the 180° backscattering is expected to be absent^8–10^. The topological protection is predicted to enhance the mobility of helical electrons and is the reason why TIs are considered for various applications^11^.

A predicted 3D topological insulator, based on strained HgTe films^5^, has been recently realized^12,13^ and a very high mobility (approaching 100 m^2^/Vs) of 2D helical electrons in this system is achieved^14,15^. The high mobility facilitates measurements of transport properties, in particular, Landau quantization of helical electrons down to low magnetic fields^12–15^ and has provided a transport verification of the non-degeneracy of the helical surface states in strained HgTe films^16^.

**Figure 1:**
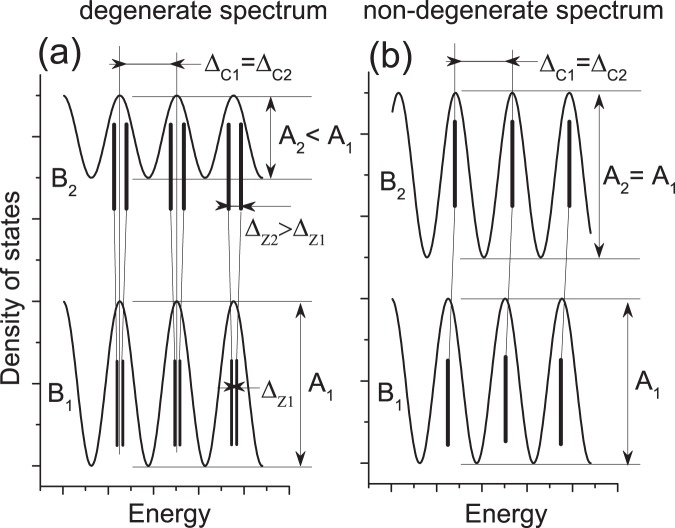
Evolution of 2D electron spectrum with an increase of the total magnetic field from ${B}_{1}$ to ${B}_{2}$ at fixed cyclotron energy: ${\Delta }_{C1}={\Delta }_{C2}$. (**a**) In spin degenerate spectrum magnetic field ${B}_{2}$ increases the spin splitting ${\Delta }_{Z2} > {\Delta }_{Z1}$ of Landau levels leading to a decrease of the amplitude of fundamental harmonic of the density of states: ${A}_{2} < {A}_{1}$ and, thus, the amplitude of SdH oscillations^17,18^. (**b**) In spin non-degenerate spectrum magnetic field $B$ does not split Landau levels and, thus, keeps the amplitude of fundamental harmonic intact: ${A}_{2}={A}_{1}$.

Below we present transport investigations of quantum resistance oscillations of highly mobile 2D helical electrons in HgTe strained films placed in tilted magnetic fields. Due to the spin-momentum locking a propagating quantum state of a 2D helical electron is non-degenerate and, thus, cannot split in a magnetic field. In contrast the spin degenerate propagating state of an ordinary 2D electron splits on spin-up and spin-down levels by the magnetic field that leads to large variations of the amplitude of Shubnikov de Haas (SdH) oscillations in tilted magnetic fields^17,18^. Figure 1 illustrates the difference between two spectra. Thus, the angular variations of SdH resistance oscillations of 2D helical electrons are not expected since the electron spin non-degenerate quantum states do not split.

Experiments presented below demonstrate that, despite the spin non-degeneracy of the electron spectrum, a tilt of the magnetic field *B* with respect to 2D layer strongly reduces the amplitude of the quantum oscillations. Mechanisms leading to the effect are not known. A phenomenological model of the effect is proposed. Comprehensive investigations of this unusual effect show that both temperature independent and temperature dependent factors are responsible for this anomalous damping of SdH oscillations of the 2D helical electrons. The temperature independent factor is consistent with a reduction of an effective quantum mean free path in magnetic fields. The temperature dependent factor indicates an increase of the reciprocal Fermi velocity ${v}_{F}^{-1}$ of 2D helical electrons in magnetic field: $\delta ({v}_{F}^{-1})\sim B$. This outcome suggests a modification of the electron spectrum $\varepsilon (\overrightarrow{k})$ and the dynamics of 2D helical electrons in magnetic fields.

## Results

**Figure 2:**
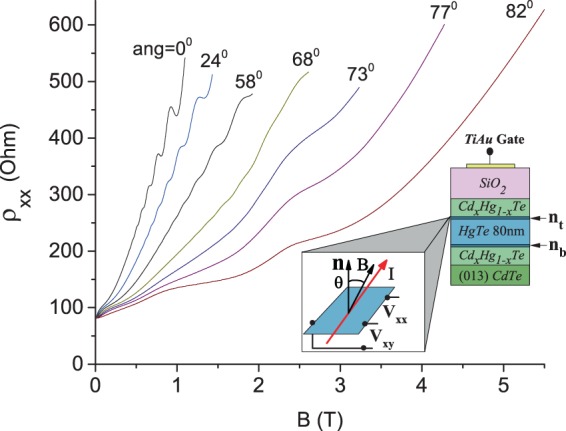
Dependence of resistivity ${\rho }_{xx}$ of 2D helical electrons on magnetic field, $B$, applied at different angles *θ* with respect to HgTe layers as labeled. Visible at *θ* = 0° oscillating content is suppressed at *θ* > 73°. The insert shows the studied structures and geometry of the experiments. Sample TI5. *V*_*g*_ = 2.5 V. T = 4.2 K.

In Fig. 2 the insert shows the studied structures and geometry of the experiments (see subsection “Experiment” in section “Methods” for detail). The top and bottom surfaces of HgTe thick film contain 2D helical electrons with density ${n}_{t}$ and ${n}_{b}$. Investigations of quantum resistance oscillations of 2D electrons located at the top surface are presented below.

Figure 2 shows the dissipative magnetoresistivity ${\rho }_{xx}(B)$ taken at different angles *θ* as labeled. Quantum resistance oscillations are visible at θ = 0°, 24° and 58° and are significantly suppressed at θ > 68°. To facilitate the analysis of the oscillating content, the monotonic background ${\rho }_{xx}^{b}(B)$, obtained by an adjacent point averaging over the period of the oscillations in reciprocal magnetic fields, is removed from the magnetoresistivity ${\rho }_{xx}(B)$.

**Figure 3:**
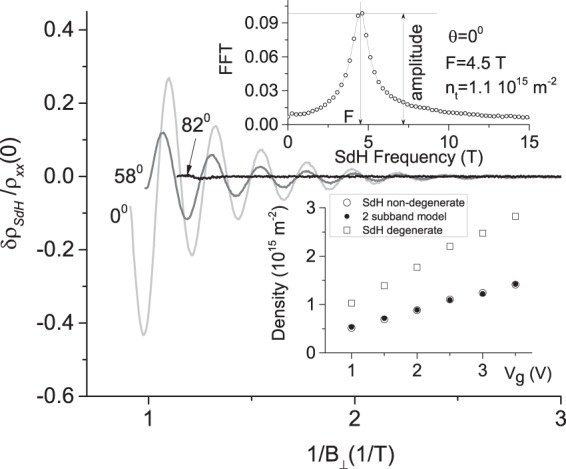
Dependence of normalized resistance oscillations $\delta {\rho }_{SdH}/{\rho }_{xx}\mathrm{(0)}$ of 2D helical electrons on reciprocal perpendicular magnetic field, ${B}_{\perp }^{-1}$, at different angles *θ* as labeled. The amplitude of the SdH oscillations reduces with the angle and is zero at *θ* = 82°. Upper insert shows FFT spectrum of the oscillations started at ${({B}_{\perp }^{-1})}^{L}$=1.09(1/T) at *θ* = 0°. Lower insert shows electron density determined by different methods. Filled circles present the density, ${n}_{1}$, obtained from comparison of the magnetoresistance with two subband model (see Fig. 9(c)). Open circles (squares) present the density determined from the frequency of SdH oscillations for spin non-degenerate (degenerate) spectrum. Sample TI5. ${V}_{g}$=2.5 V. T = 4.2 K.

Figure 3 presents the remaining oscillating content of the magnetoresistivity, $\delta {\rho }_{SdH}={\rho }_{xx}-{\rho }_{xx}^{b}$, normalized by ${\rho }_{xx}(B=\mathrm{0)}$ as a function of the reciprocal perpendicular magnetic field ${B}_{\perp }^{-1}$. As expected, the SdH oscillations are periodic in ${B}_{\perp }^{-1}$^18,19^. In agreement with Fig. 2, SdH oscillations decrease with the angle *θ* and are absent at *θ* = 82°. The upper insert shows the Fourier spectrum obtained by Fast Fourier Transformation (FFT) of the oscillations taken between $\mathrm{1/}{B}_{\perp }^{L}$ = 1.09 (1/T) and $\mathrm{1/}{B}_{\perp }^{R}$ = 5 (1/T) at *θ* = 0°. The SdH frequency *F* = 4.5(T) yields the 2D electron density ${n}_{t}=(e/h)F$ = 1.1 10^15^ m^−2^ ^18,19^. At a fixed gate voltage, ${V}_{g}$, the density ${n}_{t}$ is found to be the same at different angles *θ* indicating that the magnetic field does not change the electron density. A comparison of the density ${n}_{t}$ with the total density obtained from the Hall resistance, shown in Fig. 9(b), indicates a presence of second group of 2D electrons with a density ${n}_{b}$ = 0.8 10^15^ m^−2^. This density provides SdH oscillations at frequency 3.3 (T). These oscillations are absent in the spectrum at small ${B}_{\perp }$, which is consistent with previous experiments^14^.

In Fig. 3 the lower insert shows a comparison of the electron densities, ${n}_{1}$ and ${n}_{t}$, obtained by different methods at different gate voltages. The filled symbols present the density ${n}_{1}$, extracted from a comparison of the magnetoresistivity and Hall resistance with a two-subband model^20^. Subsection “Two subband model” of section “Methods” contains details of this analysis. Open symbols demonstrate the density, ${n}_{t}$, computed from the frequency of quantum oscillations. For a non-degenerate spectrum, ${n}_{t}=(e/h)F$, and the computations yield density presented by open circles. This density is in good agreement with the density ${n}_{1}$. For the spin degenerate electron spectrum ${n}_{t}=\mathrm{2(}e/h)F$ and computations yield density presented by open squares. This density is approximately twice as large compared to ${n}_{1}$ obtained from the two-subband model. Thus, the comparison indicates that the studied electron system has spin non-degenerate spectrum. This outcome is in accord with previous works^12–16^.

### Analysis of angular dependence

**Figure 4:**
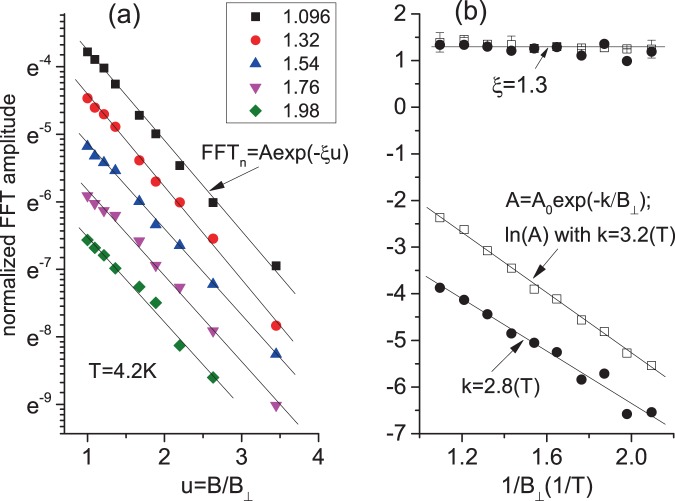
(**a**) Dependence of normalized $FFT$ amplitude of normalized resistance oscillations $\delta {\rho }_{SdH}/{\rho }_{xx}\mathrm{(0)}$ on $B/{B}_{\perp }$. $FF{T}_{n}$ amplitude is obtained for SdH oscillations in interval [${B}_{\perp }^{-1}$, 5] T^−1^. Different symbols correspond to different ${B}_{\perp }^{-1}$ as labeled. Thin straight lines are fits in accordance with Eq. (4) yielding $A$ and $\xi$. (**b**) Dependence of fitting parameters $\xi$ and $ln(A)$ on ${B}_{\perp }^{-1}$. The parameter $\xi$ = 1.3 $\pm$ 0.15 indicates uniform (${B}_{\perp }$-independent) relative decrease of SdH amplitude with angle *θ*. Open squares (filled circles) present results for $\delta {\rho }_{SdH}/{\rho }_{xx}\mathrm{(0)}$ ($\delta {\rho }_{SdH}/{\rho }_{xx}({B}_{\perp })$) normalization. Sample TI5. *V*_*g*_ = 2.5 V. T = 4.2 K.

To analyze the observed angular decrease of the amplitude of SdH oscillations in the spin non-degenerate electron system, one should assume that some physical parameters, controlling the SdH amplitude in Lifshits-Kosevich formula^18,19^, change with the magnetic field. Subsection “Model” of section “Method” contains a derivation of Lifshits-Kosevich formula and presents a logic and detail of modifications leading to the angular dependence. In Fig. 4(a) the presented data indicate an exponential decrease of the oscillations amplitude with $u=B/{B}_{\perp }=\mathrm{1/}cos(\theta )$. This property suggests that possible modifications of the parameters within the exponential Dingle, $\delta$, and temperature dependent, A(T), factors, controlling the amplitude of SdH oscillations, should be proportional to $B/{B}_{\perp }$ (see Eq. (13)). The following dependence of the effective quantum mean free path ${l}_{q}$ and Fermi velocity ${v}_{F}$ on the magnetic field B:

1 $${l}_{q}^{-1}={l}_{0}^{-1}\mathrm{(1}+\alpha B);\,{v}_{F}^{-1}={v}_{0F}^{-1}\mathrm{(1}+\beta B)$$

where ${l}_{0},{v}_{0F},\alpha ,\beta$ are constants, lead to the exponential decrease of $\delta$ and $A(T)$ with $B/{B}_{\perp }$. Derived under this assumption Eq. (17) demonstrates the exponential decrease of the amplitude of SdH oscillations with the $B/{B}_{\perp }\mathrm{=1/}cos(\theta )$.

Described by Eq. (17) small relative variations of the conductivity, $\delta {\sigma }_{SdH}$, are related to small relative variations of the resistivity, $\delta {\rho }_{SdH}$, measured in the experiment:

2 $$\delta {\sigma }_{SdH}/{\sigma }_{D}=\delta {\rho }_{SdH}/{\rho }_{N}$$

where ${\sigma }_{D}$ is classical (Drude) conductivity and ${\rho }_{N}$ is a normalizing resistivity (see subsection“Normalization” of Section “Method” for detail).

To analyze the fundamental harmonic of the resistivity, oscillating at frequency *F* in Fig.(3), we use Fast Fourier Transformation (FFT) of the normalized oscillations of the resistivity, $\delta {\rho }_{SdH}/{\rho }_{N}$ with normalization ${\rho }_{N}={\rho }_{xx}\mathrm{(0)}$. The Fourier analysis separates SdH oscillations from the top and bottom layers and/or 3D bulk (if any) exhibiting different frequencies. The experimental FFT amplitude is compared with the Fourier amplitude obtained from the Fourier transformation of the normalized oscillations of the conductivity described by Eq. (17). The Fourier analysis of Eq. (17) yields the following dominant term for the Fourier amplitude of the fundamental harmonic at frequency *F*:

3 $$FFT(\delta {\sigma }_{SdH}/{\sigma }_{D})=\frac{4aT\mathrm{[(3}\beta u+\mathrm{1/}{B}_{\perp })k+\mathrm{1]}}{{k}^{2}}\times FF{T}_{n}={C}_{n}\times FF{T}_{n}$$

where ${C}_{n}$ is a normalizing function and the normalized amplitude $FF{T}_{n}=FFT/{C}_{n}$ reads:

4 $$FF{T}_{n}(u,T,{B}_{\perp })={A}_{0}exp(-\frac{k}{{B}_{\perp }})exp(-\xi u)$$

here $d=\pi \hslash {k}_{F}/(e{l}_{0})$, $a=2{\pi }^{2}{k}_{B}{k}_{F}/(e{v}_{0F})$ and $\xi ={\xi }_{1}+{\xi }_{2}T=\alpha d+\beta aT$, $k=d+aT$, $u$ = $B/{B}_{\perp }$ = $\mathrm{1/}cos(\theta )$. ${A}_{0}=1$ is a constant. In Eq. (4) the second exponential factor describes the observed angular dependence of the SdH amplitude. The first exponent describes the usual decay of the oscillations at small magnetic fields, ${B}_{\perp }$. Below Eq. (4) is used to analyze the angular dependence of the normalized FFT amplitude of quantum oscillations of the resistivity via the relation based on Eqs. (2) and (3):

5 $$FFT(\delta {\sigma }_{SdH}/{\sigma }_{D})/{C}_{n}=FF{T}_{n}=FFT(\delta {\rho }_{SdH}/{\rho }_{N})/{C}_{n}$$

Figure 4(a) presents the dependence of the normalized *FFT* amplitude of the SdH resistance oscillations, normalized by ${\rho }_{N}={\rho }_{xx}\mathrm{(0)}$, on $u=B/{B}_{\perp }$ at different ${B}_{\perp }$ as labeled with the different symbols. The experiment indicates, that in a broad range of ${B}_{\perp }$, the SdH amplitude decreases exponentially with *u*. This result is in good agreement with Eq. (4), which is presented by thin straight lines in Fig. 4(a). The fit with Eq. (4) yields the parameter $\xi$ and amplitude *A*. Figure 4(b) shows that the extracted parameter $\xi$ is nearly independent on ${B}_{\perp }$. The obtained magnitude, *A*, drops exponentially with $\mathrm{1/}{B}_{\perp }$. This decrease is in good agreement with Eq. (4) presented by the straight thin lines: $A={A}_{0}exp(-\,k/{B}_{\perp })$. Similar results are obtained at different densities ${n}_{t}$ on both samples.

In the studied system the normalizing resistivity, ${\rho }_{N}$, is not well defined (see subsection “Normalization” for detail). Figure 4(b) indicates that the particular normalization of quantum oscillations is not essential for the angular dependence. Indeed, the extracted parameter $\xi$, describing the exponential angular decay of SdH amplitude is practically the same: $\xi =1.3\pm 0.15$ for quite different normalization: $\delta {\rho }_{SdH}/{\rho }_{xx}\mathrm{(0)}$ presented by open squares and $\delta {\rho }_{SdH}/{\rho }_{xx}^{b}({B}_{\perp })$ presented by filled circles. Here ${\rho }_{xx}^{b}({B}_{\perp })$ is the background resistivity, obtained by averaging out the oscillating content shown in Fig. 2.

The normalization affects significantly the overall amplitude of SdH oscillations, ${A}_{0}$, and quite weakly the extracted decay rate $k$. The widely used normalization by the resistivity at zero magnetic field, $\delta {\rho }_{SdH}/{\rho }_{xx}\mathrm{(0)}$, yields the following rate of the SdH decay with $\mathrm{1/}{B}_{\perp }$: $k=3.2$ and the SdH magnitude ${A}_{0}$=3.35. The normalization by the background resistivity in magnetic fields, $\delta {\rho }_{SdH}/{\rho }_{xx}^{b}({B}_{\perp })$, yields the similar decay rate: $k=2.8$ but considerably smaller SdH magnitude ${A}_{0}$ = 0.45. The magnitude ${A}_{0}$ = 0.45 is within expectations of two subband model indicating a partial contribution of the top layer to the total conductivity.

### Analysis of temperature dependence

**Figure 5:**
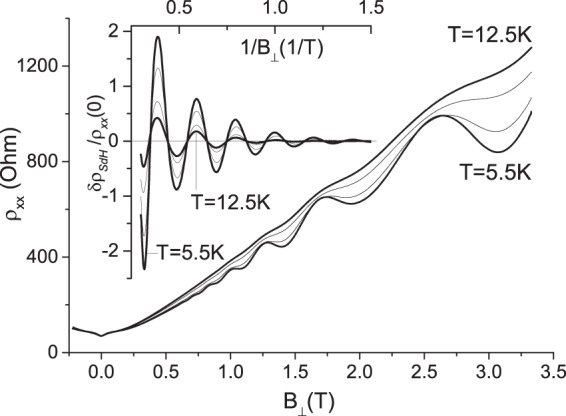
Dependence of resistivity ${\rho }_{xx}$ on magnetic field at different temperatures. From bottom to top *T* = 5.5, 7.5, 10.5 and 12.5 K. Insert shows dependence of normalized resistance oscillations $\delta {\rho }_{SdH}/{\rho }_{xx}\mathrm{(0)}$ of 2D helical electrons on reciprocal perpendicular magnetic field, ${B}_{\perp }^{-1}$, at the same set of temperatures. Sample TI1. *V*_*g*_ = 1.7 V. Angle *θ* = 0°. *n*_*t*_ = 1.2 10^15^ m^−2^.

Measurements at different temperatures reveal a temperature dependent contribution to $\xi$. Figure 5 presents the magnetic field dependence of the resistivity ${\rho }_{xx}$ at different temperatures. The insert shows the dependence of normalized resistance oscillations $\delta {\rho }_{SdH}/{\rho }_{xx}\mathrm{(0)}$ of 2D helical electrons on the reciprocal perpendicular magnetic field, ${B}_{\perp }^{-1}$, at the same set of temperatures. Figure 5 demonstrates that an increase of the temperature reduces the oscillation amplitude as expected from Eq. (17).

To analyze the temperature dependence of the SdH amplitude we rewrite Eq. (4) in the following form, separating the temperature dependent decay of SdH amplitude:

6 $$FF{T}_{n}(u,T,{B}_{\perp })={A}_{T}exp(-\,\eta T)$$

where ${A}_{T}$ = ${A}_{0}exp(-\,\alpha du-d/{B}_{\perp })$ and $\eta$ = $\beta au+a/{B}_{\perp }$. The second term in $\eta$ describes the usual exponential decay of SdH amplitude with the temperature $T$^18,19^. The first term is due to the anomalous contribution of the total magnetic field to the reciprocal velocity in Eq. (1) that leads to an additional temperature decay of SdH oscillations: ${\eta }_{0}=\beta au={\xi }_{2}u\sim B/{B}_{\perp }$.

**Figure 6:**
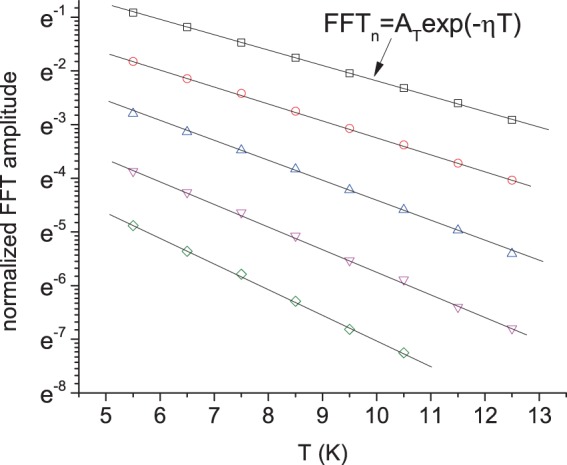
Dependence of normalized amplitude $FF{T}_{n}$ on temperature. $FF{T}_{n}$ amplitude is obtained from SdH oscillations in the interval [${B}_{\perp }^{-1}$, 3] 1/T. From top to bottom different symbols correspond to different ${B}_{\perp }^{-1}$ = 0.38, 0.59, 0.8, 1 and 1.21 1/T. Straight lines are fits, using Eq. (6) with ${A}_{T}$ and $\eta$ as fitting parameters. Sample TI1. *V*_*g*_ = 1.7 V. *n*_*t*_ = 1.2 10^15^ m^−2^.

Figure 6 presents a temperature dependence of the normalized FFT amplitude, $FF{T}_{n}$, of the normalized SdH oscillations, shown in the insert to Fig. 5. At a fixed temperature different symbols present $FF{T}_{n}$ amplitude, obtained in the interval [${B}_{\perp }^{-1}$, 3] 1/T, at different ${B}_{\perp }^{-1}$. From the top to bottom ${B}_{\perp }^{-1}$ = 0.38, 0.59, 0.8, 1 and 1.21 1/T. At a fixed ${B}_{\perp }$ the $FF{T}_{n}$ amplitude decreases exponentially with the temperature. In Fig. 6 straight lines present fits, using Eq. (6) with ${A}_{T}$ and $\eta$ as fitting parameters. Figure 6 demonstrates good agreement between the experiment and Eq. (6).

**Figure 7:**
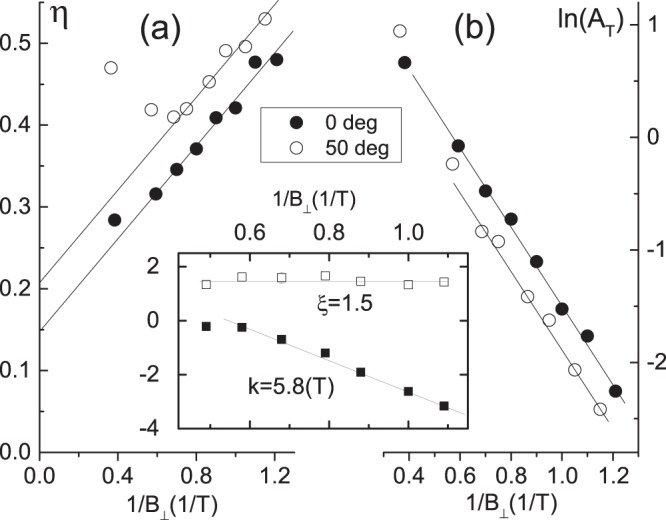
(**a**) Dependence of parameter $\eta$ obtained from the fits shown in Fig. 6 on ${B}_{\perp }^{-1}$. Straight lines present fits, using the relation $\eta =\beta au+a/{B}_{\perp }$ from Eq. (6). (**b**) Dependence of amplitude ${A}_{T}$ obtained from the fits shown in Fig. 6 on ${B}_{\perp }^{-1}$. Straight lines present fits, using the relation ${A}_{T}=exp[-(\alpha du+d/{B}_{\perp })]$ from Eq. (6). Different symbols correspond to different angles *θ* as labeled. Insert shows parameters $\xi$ and $k$ obtained from experiment at different angles *θ* and fixed temperature T = 5.5 K. Sample TI1. ${V}_{g}$ = 1.7 V. ${n}_{t}$ = 1.2 10^15^ m^−2^.

Figure 7(a) shows the dependence of the fitting parameter $\eta$ on $\mathrm{1/}{B}_{\perp }$. In Fig. 7(a) the parameter $\eta$ decreases linearly with decreasing $\mathrm{1/}{B}_{\perp }$ in good agreement with the behavior of the parameter $\eta =\beta au+a/{B}_{\perp }$ expected from Eq. (6) and presented by the thin straight lines. At $\theta$ = 0° the fit yields $a$ = 0.28 ± 0.03(T/K) and Fermi velocity ${v}_{0F}$ = 7.5(±0.8)10^5^ m/s. However, in contrast to the ordinary 2D electrons, the parameter $\eta$ does not extrapolate to zero at $\mathrm{1/}{B}_{\perp }\to 0$. Instead the comparison with Eq. (6) indicates the presence of the anomalous term ${\eta }_{0}=\beta au$ = 0.15 $\pm 0.3$ yielding $\beta$ = 0.5 ± 0.15 at u = 1 ($\theta$ = 0^0^). Taken at different angle $\theta$=50° measurements show the consistent increase of the term ${\eta }_{0}\sim u=\mathrm{1/}cos(\theta )$ with the angle: ${\eta }_{0}(u=\mathrm{1.54)}$= 0.21 ± 0.03. The presented results are obtained for the data normalization $\delta {\rho }_{SdH}/{\rho }_{xx}\mathrm{(0)}$. The normalization $\delta {\rho }_{SdH}/{\rho }_{xx}^{b}({B}_{\perp })$ yields the same *a* = 0.28 ± 0.03(T/K) and slightly higher *β* = 0.6 ± 0.15.

In Fig. 7(b) symbols present a behavior of the amplitude ${A}_{T}$, obtained from the fits shown in Fig. 6. The amplitude ${A}_{T}$ decreases exponentially with the reciprocal magnetic field, $\mathrm{1/}{B}_{\perp }$. In Fig. 7(b) the straight lines present fits, using the expression: ${A}_{T}={A}_{0}exp[-\,(\alpha du+d/{B}_{\perp })]$ for the parameter ${A}_{T}$ in Eq. (6). The fits indicate good agreement between the experiment and Eq. (6). The slope of the linear dependence $ln({A}_{T})$ vs. $\mathrm{1/}{B}_{\perp }$ yields $d$ = 3.5 $\pm 0.3$. In accordance with Eq. (17) $d=\pi \hslash {k}_{F}/(e{l}_{0})$ and, thus, at density ${n}_{t}$ = 1.2 10^15^ m^−2^ the effective quantum mean free path is ${l}_{0}$ = 73 nm in the studied sample. At $\theta$ = 50° (u = 1.54), the dependence shifts down yielding ${\xi }_{1}$ = $\alpha d$ = 0.76 $\pm$ 0.15 and $\alpha$ = 0.22 $\pm$ 0.05.

Using the obtained parameters $a,d,\alpha ,\beta$ we evaluate the parameters ${\xi }_{ev}=\alpha d+\beta aT$ = 1.58 $\pm$ 0.35 and ${k}_{ev}=d+aT$ = 5 $\pm$ 0.8 at temperature $T$ = 5.5 K The estimated parameters are close to the ones obtained in independent experiment executed at different angles and fixed temperature T = 5.5 K: $\xi$ = 1.5 ± 0.1 and $k$ = 5.8 ± 0.3. In Fig. 7 the insert shows this data. Thus, the cross examination indicates a consistency of the obtained results.

## Discussion and Possible Mechanisms

The presented above data reveal a strong suppression of SdH oscillations of 2D helical electrons in tilted magnetic fields. For the spin non-degenerate spectrum of 2D helical electrons the result is unexpected. Figure 4, Fig. 6 and Fig. 7 show good agreement between the experiments and a phenomenological model, assuming a magnetic field dependence of the quantum mean free path: ${l}_{q}^{-1}={l}_{0}^{-1}\mathrm{(1}+\alpha B)$ and Fermi velocity ${v}_{F}^{-1}={v}_{0F}^{-1}\mathrm{(1}+\beta B)$, that leads to Eqs. (4,6,17). The comparison between the model and experiment yields $\alpha$ = 0.22 $\pm$ 0.03(T^−1^) and $\beta$ = 0.5 $\pm$ 0.15(T^−1^) at ${n}_{t}$ = 1.2 10^15^ m^−2^. There is no quantitative theory of the observed anomalous angular dependence. The question regarding the dominant mechanisms leading to the observed effect is open. Below we discuss mechanisms, which may contribute to the magnetic field induced decrease of SdH oscillations.

The amplitude of SdH oscillations decreases exponentially with $u=B/{B}_{\perp }$: ${A}_{SdH}\sim exp[-\xi (B/{B}_{\perp })]$ and, thus, at a fixed ${B}_{\perp }$ with the total magnetic field $B$. The proportionality of the anomalous contributions in $\mathrm{1/}{l}_{q}$ and $\mathrm{1/}{v}_{F}$ (see Eq. (1)) to the total magnetic field suggests a possible relevance of spin effects proportional to $B$. In response to the Lorentz force, ${F}_{L}=ev\times B$, electrons in a single band move in accordance with the quasi-classical theory, considering effects of the Lorentz force on the band structure to be negligibly small^20^. In the systems with no spin-orbit interaction the $k$-space and spin $s$-space are disentangled. A change of the electron energy via Zeeman effect repopulates the spin-up and spin-down subbands in the $k$-space keeping the energy dispersion of electrons intact: ${\varepsilon }_{\uparrow }(k)$ = ${\varepsilon }_{\downarrow }(k)$. Thus at a fixed ${k}_{F}$ (electron density) both the Lorentz force and Zeeman effect should not change the Fermi velocity ${v}_{F}$. In systems with a spin-orbit coupling a variation in the $s$-space via the Zeeman term, may change the electron dispersion in the $k$-space and lead to a variation of the electron velocity ${v}_{F}$. To illustrate this effect we consider a simple model of 2D helical electrons affected by the Zeeman term $\Delta \sim B=\mathrm{(0,0,}{B}_{z})$. The following Hamiltonian describes 2D helical states of a 3D topological insulator (see Eq. (34) in ref. ^9,21^

7 $$H=C+E({\sigma }^{x}{k}_{y}-{\sigma }^{y}{k}_{x})+\Delta {\sigma }^{z}$$

where $C$ and $E$ are material constants, ${\sigma }^{x,y,z}$ are Pauli matrices and ${\rm{k}}=({k}_{x},{k}_{y})$ is the 2D electron wave vector. The Zeeman term $\varDelta {\sigma }^{z}$ changes the electron spectrum leading to a spectral gap:

8 $$\varepsilon (k)=C\pm {({\varDelta }^{2}+{E}^{2}{k}^{2})}^{\mathrm{1/2}}$$

**Figure 8:**
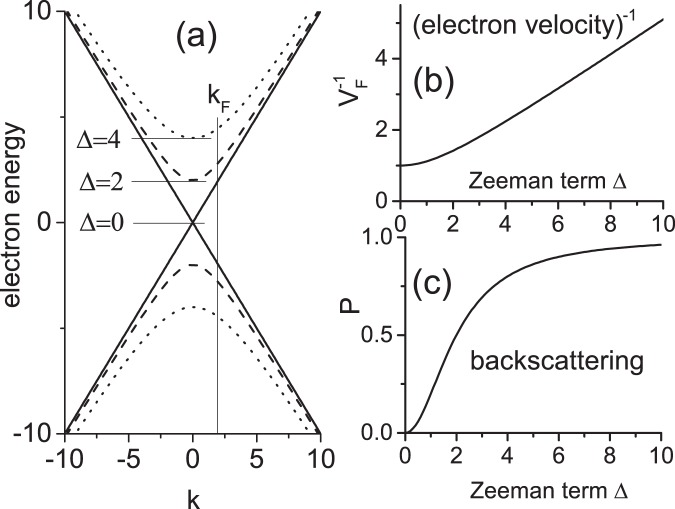
(**a**) Counted from $C$ energy dispersion $\varepsilon (k)$ of 2D helical electrons at different values of Zeeman energy Δ as labeled; (**b**) variations of reciprocal Fermi velocity with Zeeman energy; (**c**) variations of normalized probability $P$ of electron backscattering with Zeeman energy. *E* = 1; ${k}_{F}$ = 2. All parameters are in relative units.

Figure 8(a) presents the electron spectrum at different strengths of the Zeeman term as labeled and *E* = 1. The vertical thin line indicates the electron wave number ${k}_{F}$ at Fermi energy. Figure 8(b) shows the increase of the reciprocal Fermi velocity ${v}_{F}^{-1}=(\partial \varepsilon /\partial k{)}^{-1}$($k$ = ${k}_{F}$) with Δ, following from Eq. (8). The increase is proportional to *B* at a large Δ.

This simple model also exhibits an increase of the electron scattering in magnetic fields. By polarizing electron spins in the *z*-direction the magnetic field increases the spin overlap between incident ${k}_{F}({\theta }_{in})$ and scattered ${k}_{F}({\theta }_{fin})$ electron states. Figure 8(c) presents the dependence of a normalized rate of the electron backscattering (${\theta }_{fin}-{\theta }_{in}=\pi )$ on the Zeeman term $\Delta \sim B$. The presented probability is a square of the magnitude of the scalar product of two eigenvectors of Hamiltonian (7) corresponding to incident ${k}_{F}({\theta }_{in})$ and scattered ${k}_{F}({\theta }_{fin})$ electron states. At $\Delta \mathrm{=0}$ the rate is zero indicating the topological protection of the backscattering. With increasing Δ the rate increases imitating a linear dependence on Δ in the interval from 0.5 to 2. At high Δ the rate approaches 1 indicating that at high magnetic fields there are no spin restrictions on the impurity scattering since all electron spins are polarized along $B$.

The presented illustrative model demonstrates variations of both the reciprocal velocity, $\mathrm{1/}{v}_{F}$, and the scattering time $\mathrm{1/}\tau$ of 2D helical electrons with the total magnetic field, which are qualitatively similar to variations of the corresponding parameters, observed in the experiments. However, some properties of the model indicate an inconsistency with the experiment. An estimation of the magnitude of the Zeeman energy, Δ, for HgTe quantum well yields $\Delta \sim$ 1 meV at *B* = 1 T. This value is of an order of magnitude smaller than both the energy gap ${\Delta }_{g}\approx$ 15 meV and the Fermi energy of 2D helical electrons ${E}_{F}=\hslash {v}_{F}{k}_{F}\sim$ 60 meV. These estimations suggest a rather weak effect of the Zeeman energy on the electron spectrum. Furthermore, for a linear spectrum the Zeeman term, produced by an in-plane magnetic field, is found to be ineffective. This term shifts the energy spectrum in the xy-plane of the k-space, but does not change the Fermi velocity and the backscattering. Recent theoretical investigations indicate, however, that an account of nonlinear momentum terms in the TI Hamiltonian leads to a tilt of the Dirac cone by in-plane magnetic fields^22^. The tilt of the cone may increase the electron backscattering and, thus, may contribute to the anomalous decay presented in this report. The studied 2D helical electrons are result of a linear superposition of electron states from several subbands and additional terms may also affect the spectrum^23^. A quantitative comparison with the experiment requires a development of more realistic models and is beyond of this report.

Presented at the end of the section “Results” data analysis indicates a short effective quantum mean free path of electrons: ${l}_{q}\sim$ 100 nm. A comparison of the positive magnetoresistance with the two-subband model, presented in section “Methods”, yields the transport mean free path, ${l}_{tr}$, which is about few microns. Such a large difference between two lengths suggests either dominant contribution of a very small angular scattering of electrons or a strong in-homogeneous broadening of the SdH oscillations or both effects. The small angular scattering is a general property of highly mobile 2D electron systems with a remote doping^24^. In the studied electron system, however, the remote doping is absent. The spin restrictions for the electron-impurity scattering, due to the spin-momentum locking for 2D helical electrons, do not provide a large difference between the transport and quantum mean free times of 2D helical electrons since $\mathrm{1/}{\tau }_{q}(\varphi )\sim \mathrm{1/}{\tau }_{tr}(\varphi )$ for the most of scattering angles $\varphi$ except an angular sector around $\varphi =\pi$ (see Fig. 8(c)). Thus the large difference between ${l}_{q}$ and ${l}_{tr}$ points toward a presence of a substantial in-homogeneous broadening of the SdH oscillations.

A possible reason of the in-homogeneous broadening is a spatial non-uniformity and/or fluctuations of the mechanical strain, induced by the intentional lattice mismatch between HgTe film and CdTe substrate. The strain is anticipated to be non-uniform in the direction perpendicular to the boundary between HgTe and CdTe substrate since at a large distance the strain should relax. In the lateral direction the strain may fluctuate due to growth defects such as dislocations or growth steps.

The induced mechanical strain creates the insulating gap ${\Delta }_{g}$ in the bulk of the HgTe film, which is, thus, spatially non-uniform: ${\Delta }_{g}(r)$. Since the 2D helical electron state is a linear combination of electron and hole states from the conduction and valence band of the bulk 3D insulator^9^ the gap ${\Delta }_{g}(r)$ affects both the spectrum and the density of 2D helical electrons. The spatial variations of the gap lead to spatial variations of the spectrum and the electron density resulting in the in-homogeneous broadening of SdH oscillations.

To produce the observed magnetic field response the spatial dispersion of the strain magnitude should increase with the magnetic field, $B$. A possible mechanism, which may lead to such increase, is the effect of magnetostriction^25^. The magnetostriction induces a mechanical strain of materials upon application of a magnetic field. The effect is strong in ferromagnetic metals since a substantial part of electrons contribute to the magnetization, and, thus, to the free energy in magnetic field. In contrast in nonmagnetic normal metals, due to the spin degeneracy of electron spectrum, only a small part of electrons ($\varDelta n/n\approx {\varDelta }_{Z}/{E}_{F}\sim$ 1) contribute to the magnetization^20^ and the magnetostriction is small. 2D helical electrons have the spin non-degenerate spectrum and, thus, similarly to the ferromagnetic metals, should all contribute to the magnetization, enhancing the magnetostriction effect.

## Summary

In summary, the angular dependence of quantum resistance oscillations of 2D helical electrons in 3D topological insulators, based on strained HgTe films, demonstrates exponentially strong reduction of the oscillation amplitude $A$ in tilted magnetic field $B$: $A\sim exp[-(\xi /{B}_{\perp })B]$. The temperature dependence of the amplitude $A$ reveals two terms contributing to the parameter $\xi ={\xi }_{1}+{\xi }_{2}T$. The temperature independent term, ${\xi }_{1}$, indicates considerable reduction of the effective quantum mean free path ${l}_{q}$ in the magnetic field $B$. The reduction is consistent with the form: $[\delta ({l}_{q}^{-1})]/{l}_{0}^{-1}$ = $\alpha B$, where $\alpha$ = 0.22 $\pm$ 0.03(T^−1^) at electron density *n*_*t*_ = 1.2 10^15^ m^−2^. A suppression of the topological protection of the helical electron states against the impurity scattering in magnetic fields may contribute to the effect. Observed large difference between the effective quantum and transport mean free paths points toward mechanisms, leading to an increase of in-homogeneous broadening of SdH oscillations in magnetic fields. The temperature dependent term, ${\xi }_{2}T$, is consistent with an increase of the reciprocal velocity ${v}_{F}^{-1}$ of 2D helical electrons in the magnetic field: $[\delta {v}_{F}^{-1}]/{v}_{0F}^{-1}=\beta B$, where $\beta$ = 0.5 $\pm$ 0.15(T^−1^) at ${n}_{t}$ = 1.2 10^15^ m^−2^. This increase suggests a modification of the dynamics of 2D helical electrons in the magnetic field.

## Methods

### Experiment

Studied, 80 nm wide, strained HgTe films are grown by molecular beam epitaxy on (0,1,3) CdTe substrate. Since HgTe films grown directly on CdTe suffer from dislocations due to the lattice mismatch, our 80 nm thick HgTe films were separated from the CdTe substrate by a 20 nm thin Cd_0.7_ Hg_0.3_ Te buffer layer. This buffer layer significantly increases the electron mobility up to 40 m^2^/(Vs)^14^. In Fig. 2 the insert shows the studied structures. The 2D helical electrons are formed at the top and the bottom surfaces of the HgTe film. The structures are equipped with a TiAu gate providing the possibility to tune the Fermi energy ${E}_{F}$ inside the insulating gap ${\varDelta }_{g}\approx$ 15 mV^14^ and to change the density $n={n}_{t}+{n}_{b}$ of 2D helical electrons, where ${n}_{t}$ (${n}_{b}$) is the density of 2D electrons located at the top (bottom) of HgTe film. Magnetotransport experiments indicate that at a large positive gate voltage ${V}_{g}$, ${n}_{t} > {n}_{b}$ since the top HgTe surface is closer to the gate^14^. Reported in this paper measurements are done, when Fermi energy is inside the gap ${\Delta }_{g}$.

Samples are etched in the shape of a Hall bar with width $W=50\mu$m. Two samples are studied in magnetic fields up to 8 Tesla applied at different angle *θ* relative to the normal *n* to 2D layers and perpendicular to the applied current. The angle *θ* is evaluated using Hall resistance *R*_*xy*_, which is proportional to the perpendicular component, ${B}_{\perp }=Bcos(\theta )$, of the total magnetic field *B*. Experiments indicate that 2D helical electrons located at the top of HgTe film provide the dominant contribution to SdH oscillations at small magnetic fields^14,15^. The density *n*_*t*_ is estimated from the frequency of SdH oscillations taken at *θ* = 0° (see upper insert to Fig. 3) and from a comparison of the observed positive magnetoresistance with a two-subband model. Both methods yield very consistent results for the electron density, *n*_*t*_, shown by the circles in the lower insert to Fig. 3. An averaged mobility obtained from Hall resistance and the resistivity at zero magnetic field for sample TI1 (TI5) is *μ* = 43 m^2^/Vs (37 m^2^/Vs). Sample resistance was measured using the four-point probe method. We applied a 133 Hz *ac* excitation ${I}_{ac}$ = 0.5 *μ* A through the current contacts and measured the longitudinal (in the direction of the electric current, *x*-direction) and Hall (along *y*-direction) voltages. The measurements were done in the linear regime in which the voltages are proportional to the applied current.

### Model

To analyze the observed decrease of the amplitude of SdH oscillations in a spin non-degenerate electron system, one should assume that some physical parameters, controlling the SdH amplitude in Lifshits-Kosevich formula^18,19^, change with the magnetic field. We start with a derivation of the standard formula for the amplitude of the fundamental harmonic of the quantum oscillations for the spin degenerate case and a parabolic spectrum: $\varepsilon ={\hslash }^{2}{k}^{2}\mathrm{/2}m$, where $m$ is an effective mass.

In the case of small quantizing magnetic fields ${\omega }_{c}{\tau }_{q}\, < \,1$, where ${\omega }_{c}=e{B}_{\perp }/m$ is cyclotron frequency and ${\tau }_{q}$ is quantum scattering rate, the main contribution to SdH oscillations comes from the fundamental harmonic of quantum oscillations of the density of states (DOS) corresponding to spin-up and spin-down subbands. The total DOS, $\nu (\varepsilon )$, reads^18^:

9 $$\nu (\varepsilon )={\nu }_{0}\left[1-\delta cos\left(\frac{2\pi (\varepsilon -{\Delta }_{Z}\mathrm{/2)}}{\hslash {\omega }_{c}}\right)-\delta cos\left(\frac{2\pi (\varepsilon +{\Delta }_{Z}\mathrm{/2)}}{\hslash {\omega }_{c}}\right)\right]={\nu }_{0}\left[1-2\delta cos\left(\frac{2\pi \varepsilon }{\hslash {\omega }_{c}}\right)cos\left(\frac{\pi {\Delta }_{Z}}{\hslash {\omega }_{c}}\right)\right]$$

where $\delta =exp(\,-\,\pi /{\omega }_{c}{\tau }_{q})$ is Dingle factor, ${\nu }_{0}$ is the total DOS at zero magnetic field, ${\Delta }_{Z}=\mu gB$ is Zeeman energy and $g$ is $g$-factor. Equation (9) indicates that the amplitude of the fundamental harmonic is controlled by the spin dependent factor $p=cos(\pi {\Delta }_{Z}/\hslash {\omega }_{c})$. An evolution of the total (spin-up and spin-down) DOS with the magnetic field is shown in Fig. 1(a). At a fixed cyclotron energy, $\hslash {\omega }_{c}$, the amplitude of DOS oscillations decreases with the total magnetic field $B$ due to a destructive interference of DOS oscillations of spin-up and spin-down subbands, decreasing the spin dependent factor $p$ in Eq. (9). At a critical angle corresponding to ${\Delta }_{Z}=\hslash {\omega }_{c}\mathrm{/2}$ the spin dependent factor $p$ = 0 and the amplitude of the fundamental harmonic of DOS is zero.

The 2D conductivity $\sigma$ is obtained from the following relation:

10 $$\sigma (B)=\int \sigma (\varepsilon )\left(-\frac{\partial f}{\partial \varepsilon }\right)d\varepsilon =\langle \sigma (\varepsilon )\rangle $$

The integral is an average of the conductivity $\sigma (\varepsilon )$ taken essentially for energies $\varepsilon$ inside the temperature interval $kT$ near Fermi energy, where $f(\varepsilon )$ is the electron distribution function at the temperature *T*^18^. The brackets represent this integral below.

The following expression approximates the conductivity $\sigma (\varepsilon )$ at small quantizing magnetic fields^26,27^:

11 $$\sigma (\varepsilon ,{B}_{\perp },{\Delta }_{Z})={\sigma }_{D}({B}_{\perp })\tilde{\nu }{(\varepsilon ,{B}_{\perp },{\Delta }_{Z})}^{2}$$

where ${\sigma }_{D}({B}_{\perp })$ is Drude conductivity in magnetic field ${B}_{\perp }$^20^ and $\tilde{\nu }(\varepsilon )=\nu (\varepsilon )/{\nu }_{0}$ is normalized total density of states.

A substitution of Eq. (11) and Eq. (9) into Eq. (10) yields an additional term to the Drude conductivity, $\delta {\sigma }_{SdH}$, describing quantum oscillations of conductivity:

12 $$\frac{\delta {\sigma }_{SdH}}{{\sigma }_{D}}=-4\delta \langle cos\left(\frac{2\pi \varepsilon }{\hslash {\omega }_{c}}\right)\rangle cos\left(\frac{\pi {\Delta }_{Z}}{\hslash {\omega }_{c}}\right)=-\,4\delta A(T)cos\left(\frac{2\pi {\varepsilon }_{F}}{\hslash {\omega }_{c}}\right)cos\left(\frac{\pi {\Delta }_{Z}}{\hslash {\omega }_{c}}\right)$$

where ${\varepsilon }_{F}$ is Fermi energy and $A(T)=\frac{\mathrm{(2}{\pi }^{2}{k}_{B}T/\hslash {\omega }_{c})}{sinh\mathrm{(2}{\pi }^{2}{k}_{B}T/\hslash {\omega }_{c})}$ is SdH temperature factor^19^. Due to the presence of the spin factor $p=cos(\pi {\varDelta }_{Z}/\hslash {\omega }_{c})$ the SdH amplitude depends substantially on the ratio between Zeeman and cyclotron energies. In 2D electron systems this ratio varies with the angle $\theta$: ${\Delta }_{Z}/{\Delta }_{C}\sim B/{B}_{\perp }\mathrm{=1/}cos(\theta )$ since the cyclotron energy depends on the perpendicular magnetic field ${B}_{\perp }$, while the Zeeman energy is proportional to the total magnetic field $B$. It leads to the angular variations of the amplitude SdH oscillations in 2D electron systems^17,18^.

For a spin non-degenerate spectrum Eq. (9) contains only one oscillating term. Below we use the term with positive Zeeman energy yielding the following expression for SdH oscillations:

13 $$\frac{\delta {\sigma }_{SdH}}{{\sigma }_{D}}=-\,4\delta A(T)cos\left(\frac{2\pi ({\varepsilon }_{F}+{\Delta }_{Z}\mathrm{/2)}}{\hslash {\omega }_{c}}\right)=-\,4\delta A(T)cos\left(\frac{2\pi n}{{n}_{L}}+\varphi \right)$$

Due to the presence of only one spin subband, the spin factor p = 1 and, in contrast to Eq. (12), Eq. (13) does not exhibit the standard angular dependence^17,18^. In the last part of the equation we have substituted ${\varepsilon }_{F}/(\hslash {\omega }_{c})$ by $n/{n}_{L}$, where $n$ is electron density and ${n}_{L}=e{B}_{\perp }\mathrm{/2}\pi \hslash$ is the orbital degeneracy of a Landau level^20^. This substitution allows to use this formula for 2D electrons with a general spectrum. The substitution yields the correct relation between the electron density, $n$, and the SdH frequency, $F$: $n=(e/h)F$^18,20^, which has been used to find the electron density shown in the lower insert to Fig. 2. We have also introduced a phase of the SdH oscillations, $\varphi$. In addition to the Zeeman effect contribution, the phase may contain contributions from other properties of the electron spectrum such as Berry phase correction, which have been ignored in Eq. (9).

Below we consider possible modifications of the Eq. (13), which may lead to angular variations of the SdH amplitude at a fixed ${B}_{\perp }$. There are several parameters in Eq. (13), which affect the amplitude of the SdH oscillations. One of the parameters is the Dingle factor $\delta =exp(\,-\,\pi /{\omega }_{c}{\tau }_{q})$. This parameter may vary with the angle $\theta$ if the cyclotron frequency ${\omega }_{c}$ or quantum scattering rate $\mathrm{1/}{\tau }_{q}$ or both change with the total magnetic field *B* or with the component of the magnetic field parallel to 2D layer, *B* _par_ . The SdH temperature factor $A(T)$ may change if ${\omega }_{c}$ depends on *B* or *B* _par_ . Finally spatial fluctuations of Fermi energy, ${\varepsilon }_{F}$, cyclotron frequency, ${\omega }_{c}$ and/or SdH phase $\varphi$ may lead to a destructive interference of the SdH oscillations from different parts of a sample resulting in, so called, in-homogeneous broadening of Landau levels^28,29^. If the in-homogeneous broadening depends on $B$ and/or *B* _par_ , then the amplitude of SdH oscillations may depend on the angle. Variations of the described physical parameters lead to the angular variations of SdH amplitude. Below these variations are accounted via magnetic field dependent contributions to Dingle, $\delta$, and temperature dependent, $A(T)$, factors.

We use the following expression for the cyclotron frequency:

14 $${\omega }_{c}=\frac{e{v}_{F}{B}_{\perp }}{\hslash {k}_{F}}$$

This relation follows from the semi-classical equation of the electron motion in the magnetic field, ${B}_{\perp }$^20^. To simplify the analysis of the Dingle factor, we re-write this factor $\delta =exp(\,-\,\pi /{\omega }_{c}{\tau }_{q})$ in term of a quantum mean free path ${l}_{q}={v}_{F}{\tau }_{q}$:

15 $$\delta =\exp \left(\,-\,\frac{\pi \hslash {k}_{F}}{e{l}_{q}{B}_{\perp }}\right)$$

where ${k}_{F}={\mathrm{(4}\pi {n}_{t})}^{\mathrm{1/2}}$ is the electron wave number and ${v}_{F}$ is electron velocity at Fermi energy. FFT analysis indicates that the SdH frequency $F$, shown in Fig. 3, and, thus, ${n}_{t}$ and ${k}_{F}$ do not depend on the angle $\theta$. Thus, the Eq. (15) is more convenient for further analysis, since only one material parameter: ${l}_{q}$ depends on $\theta$ ($B$). Below we assume that the ${l}_{q}$ is an effective parameter containing contributions from both the impurity scattering and in-homogeneous broadening^28^.

The SdH temperature factor $A(X)=X/sin\,h(X)$, where $X=2{\pi }^{2}{k}_{B}T/\hslash {\omega }_{c}=2{\pi }^{2}{k}_{B}T{k}_{F}/(e{v}_{F}{B}_{\perp })$. At $X\mathrm{ > 1}$, corresponding to our experiments at small magnetic fields, the factor $A(X)=X/sinh(X)\approx 2Xexp(\,-\,X)$ decreases exponentially with $\mathrm{1/}{B}_{\perp }$. A modification of the Fermi velocity, ${v}_{F}$, with $B$ may lead to variations of the factor $A(T)$.

In Fig. 4(a) the presented data indicate an exponential decrease of the SdH oscillations amplitude with $B/{B}_{\perp }$. This property suggests that the possible modifications of the parameters within the exponential Dingle and temperature dependent factors should be proportional to $B/{B}_{\perp }$. The following relations of the effective quantum mean free path ${l}_{q}$ and Fermi velocity ${v}_{F}$ with the magnetic field B lead to the required exponential decrease of $\delta$, $A(T)$ and, thus, SdH amplitude with $B/{B}_{\perp }$:

16 $${l}_{q}^{-1}={l}_{0}^{-1}\mathrm{(1}+\alpha B);\,{v}_{F}^{-1}={v}_{0F}^{-1}\mathrm{(1}+\beta B)$$

where ${l}_{0},{v}_{0F},\alpha ,\beta$ are constants, Indeed, a substitution of the relations (16) into Eq. (9), Eq. (11) and Eq. (13) yields the following expression for the amplitude of SdH oscillations:

17 $$\frac{\delta {\sigma }_{SdH}}{{\sigma }_{D}}\approx -\mathrm{8(1}+3\beta B)\left(\frac{aT}{{B}_{\perp }}\right)exp\left(-\frac{d+aT}{{B}_{\perp }}\right)exp\left(-\frac{\alpha d+\beta aT}{cos(\theta )}\right)cos\left(\frac{2\pi F}{{B}_{\perp }}+\varphi \right)$$

where $d=\pi \hslash {k}_{F}/(e{l}_{0})$, $a=2{\pi }^{2}{k}_{B}{k}_{F}/(e{v}_{0F})$. In the derivation of the result we have approximated the temperature dependent factor $A(X)=X/sinh(X)\approx 2Xexp(-\,X)$ for $X\mathrm{ > 1}$. We have assumed also that $\beta B <  < 1$. Variations of the reciprocal Fermi velocity with magnetic field in Eq. (16) leads to variations of the density of states $\nu ({\varepsilon }_{F})$, since $\nu ({\varepsilon }_{F})={k}_{F}\mathrm{/(2}\pi {v}_{F})$. In Eq. (9) the DOS ${\nu }_{0}$ is replaced by ${\nu }_{0}\mathrm{(1}+\beta B)$.

In Eq. (17) the first exponential factor describes the usual decay of SdH amplitude with $\mathrm{1/}{B}_{\perp }$: parameters $d$ and $a$ are coming from the Dingle and temperature damping factors of the SdH amplitude^18,27^. The second exponential factor describes the angular variations of the SdH amplitude of 2D helical electrons. This factor leads to the exponential decrease of SdH amplitude with $B/{B}_{\perp }\mathrm{=1/}cos(\theta )$. At $\alpha =\beta =$ 0 the angular variations of the SdH amplitude are absent and expression (17) reduces to the standard Eq. (13) with ${l}_{q}={l}_{0}$ and ${v}_{F}={v}_{0F}$.

### Normalization

Quantitative analysis of SdH oscillations is based on the relation between Eqs. (12,17) and relative variations of the resistivity measured in experiments:

18 $$\delta {\sigma }_{SdH}/{\sigma }_{D}({B}_{\perp })=\delta {\rho }_{SdH}/{\rho }_{N}$$

where ${\rho }_{N}$ is a normalizing resistivity. In strong magnetic fields, at which the Hall resistivity, ${\rho }_{xy}$, is much larger the longitudinal resistivity, ${\rho }_{xx}$, the longitudinal conductivity, ${\sigma }_{xx}$ is proportional to the resistivity ${\rho }_{xx}$: ${\sigma }_{xx}=(en/{B}_{\perp }{)}^{2}{\rho }_{xx}$, where $n$ is carrier density^20^ This property leads to a relation

19 $$\delta {\sigma }_{SdH}/{\sigma }_{xx}=\delta {\rho }_{SdH}/{\rho }_{xx}$$

where $\delta {\sigma }_{SdH}$ ($\delta {\rho }_{SdH}$) is a quantum contribution to the conductivity ${\sigma }_{xx}$ (resistivity ${\rho }_{xx}$). In the simplest case of a single group of carriers the classical (Drude) resitivity does not depend on the magnetic field^20^: ${\rho }_{xx}({B}_{\perp })={\rho }_{xx}\mathrm{(0)}$ and the above relation yields:

20 $$\delta {\sigma }_{SdH}/{\sigma }_{D}({B}_{\perp })=\delta {\rho }_{SdH}/{\rho }_{xx}\mathrm{(0)}$$

where ${\sigma }_{D}({B}_{\perp })$ is Drude conductivity used in Eq. (12) for the conductivity of the single group of carriers. Equation (20) provides the relation between oscillations in the conductivity, which are evaluated theoretically, and the oscillations of the resistivity, which are measured in experiments for systems with single group of carriers.

If several groups of carriers contribute to the conductivity, as in the studied case, the situation is less certain. The reason of the uncertainty is the lack of a direct relation between the Drude conductivity ${\sigma }_{D}({B}_{\perp })$, used in Eq. (17) for a single group of carriers, and the measured resistivity ${\rho }_{xx}$, which contains contributions from several groups of carriers. In Eq. (17) the conductivity ${\sigma }_{D}({B}_{\perp })$ is the conductivity, ${\sigma }_{t}$, of electrons at the top conducting surface of the HgTe layer. The total conductivity, ${\sigma }_{tot}({B}_{\perp })={\sigma }_{t}+{\sigma }_{b}+{\sigma }_{vol}$, is a sum of conductivities of the top and bottom (${\sigma }_{b}$) surfaces and, possibly, the bulk of the film (${\sigma }_{vol}$). Thus, the $\delta {\sigma }_{SdH}/{\sigma }_{t}$ is not equal to $\delta {\rho }_{SdH}/{\rho }_{xx}({B}_{\perp })$ for the studied system with several groups of carriers. It leads to an uncertainty of the normalizing resistivity, ${\rho }_{N}$, in Eq. (18).

We have investigated the effect of the resistance normalization on results of the analysis of the angular dependence of the SdH oscillations. For very different normalizing resistance: ${\rho }_{N1}={\rho }_{xx}\mathrm{(0)}$ and ${\rho }_{N2}={\rho }_{xx}({B}_{\perp })$ we have found no difference in the extracted parameter $\xi$ controlling the angular dependence (see Fig. 4(b)). It provides a confidence that the obtained parameter $\xi$ is quantitatively correct. In addition we have found that the normalization ${\rho }_{N2}={\rho }_{xx}({B}_{\perp })$ provides an amplitude of the SdH oscillations, which is more consistent with our model.

### Two subband model

Presented in Fig. 2 data demonstrate a positive magnetoresitance. Usually the positive magnetoresistance indicates a presence of two or more groups of carriers^20^. In this section we compare the positive magnetoresistance with two subband model. In this model we assume that two groups of carriers are located at top and the bottom surfaces of the HgTe film as shown in the insert to Fig. 2. In contrast to the regular quantum wells with two populated subbands^30–32^, these two conducting 2D layers are separated from each other and do not interact. This is supported by the fact, that the magneto-inter-subband oscillations, induced by the electron inter-subband scattering^30,33,34^, are absent in the studied system. This allows us to use a simplified version of the two subband model^20^ ignoring the inter-subband scattering^35^.

We compare the two-subband model with experiments at $\theta$ = 0°. The model considers two groups of non-interacting electrons in a perpendicular magnetic field, B. Each group has electron density ${n}_{i}$, mobility ${\mu }_{i}$ and conductivity ${\sigma }_{i}=e{n}_{i}{\mu }_{i}$ at *B* = 0 T, where the index *i* = 1,2 labels each group. In a magnetic field the total Drude conductivity ${\sigma }_{xx}$ and Hall conductivity, ${\sigma }_{xy}$, read^20^:

21 $${\sigma }_{xx}=\frac{{\sigma }_{1}}{1+{({\mu }_{1}B)}^{2}}+\frac{{\sigma }_{2}}{1+{({\mu }_{2}B)}^{2}};\hspace{0.5cm}{\sigma }_{xy}=\frac{{\sigma }_{1}{\mu }_{1}B}{1+{({\mu }_{1}B)}^{2}}+\frac{{\sigma }_{2}{\mu }_{2}B}{1+{({\mu }_{2}B)}^{2}}$$

The longitudinal resistivity, ${\rho }_{xx}$, and Hall resistance, ${R}_{xy}={\rho }_{xy}$, are obtained by the inversion of the conductivity matrix:

22 $${\rho }_{xx}=\frac{{\sigma }_{xx}}{{({\sigma }_{xx})}^{2}+{({\sigma }_{xy})}^{2}};\hspace{0.5cm}{\rho }_{xy}=\frac{{\sigma }_{xy}}{{({\sigma }_{xx})}^{2}+{({\sigma }_{xy})}^{2}}$$

**Figure 9:**
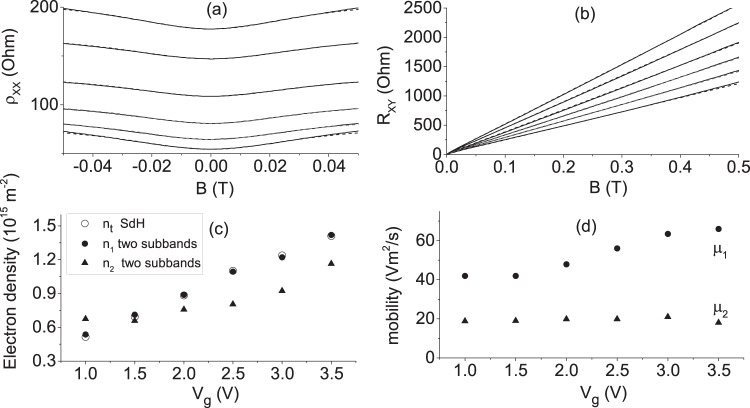
(**a**) Dependence of the longitudinal resistivity, ${\rho }_{xx}$ and (**b**) dependence of the Hall resistance, ${R}_{xy}$, on the magnetic field, B, directed perpendicular to the 2D plane. Different curves present dependencies taken at different gate voltages, ${V}_{g}$. From the top to the bottom: ${V}_{g}$ is from 1 V to 3.5 V with step 0.5 V. Solid (dash) lines present experimental data (fits, using two subband model); (**c**) filled symbols present electron densities in two subbands, ${n}_{1}$ and ${n}_{2}$, extracted from the fits shown in (**a**,**b**). Open circles present electron density obtained from the frequencies of SdH oscillations ${n}_{SdH}=(e/h)F$; (**d**) Electron mobility, ${\mu }_{1}$ and ${\mu }_{2}$, in two subbands, extracted from the fits shown in (**a**) and (**b**). Sample TI5. T = 4.2 K.

Figure 9(a,b) present a comparison of the resistivity, ${\rho }_{xx}$, and Hall resistance, ${R}_{xy}={\rho }_{xy}$, with the two subband model. Solid lines demonstrate the experimental data, while the dashed lines are computed, using Eq. (21) and Eq. (22). The electron density, ${n}_{i}$, and mobility, ${\mu }_{i}$, are fitting parameters for the computations of ${\rho }_{xx}$ and ${R}_{xy}$. A good agreement is found between ${\rho }_{xx}(B)$ and the model at magnetic fields below 0.05 T. At the same fitting parameters the Hall resistance follows the two subband model for larger magnetic fields.

Figure 9(c) presents a dependence of the electron densities ${n}_{1}$ and ${n}_{2}$, obtained from the fit shown in Fig. 9(a,b), on the gate voltage ${V}_{g}$. The top layer with density ${n}_{t}$ is related to the electron group with density ${n}_{1}$: ${n}_{t}={n}_{1}$. The top layer is located closer to the gate and, thus, more sensitive to the gate voltage variations. This layer provides an additional screening for the bottom layer that leads to the weaker dependence of the electron density ${n}_{b}={n}_{2}$ on ${V}_{g}$. Open circles present the density obtained from the frequency $F$ of SdH oscillations using spin non-degenerate spectrum: ${n}_{SdH}=(e/h)F$. For a broad range of gate voltages there is a good agreement between density ${n}_{1}$ and ${n}_{SdH}$. This agreement indicates that the spectrum of 2D electrons in the top layer is non-degenerate.

Figure 9(d) presents a dependence of the mobility ${\mu }_{i}$ on the gate voltage ${V}_{g}$. The mobility at the bottom layer is found to be ${\mu }_{2}\approx$ 20 (Vm^2^/s) and is weakly dependent on ${V}_{g}$. The mobility at the top layer, ${\mu }_{1}$ has a higher value and exhibits a considerable increase at high ${V}_{g}$. Below we evaluate the transport mean free path, ${l}_{tr}$, of the electrons. The mobility $\mu =e{\tau }_{tr}/m$, where ${\tau }_{tr}$ is a transport mean free time, can be rewritten in the following form $\mu =\frac{e{l}_{tr}}{\hslash {k}_{F}}$, where ${l}_{tr}={v}_{F}{\tau }_{tr}$ and $\hslash {k}_{F}=m{v}_{F}$. For the transport mean free path we found ${l}_{tr}^{\mathrm{(1)}}\approx$ 4.4 microns (${l}_{tr}^{\mathrm{(2)}}\approx$ 1.3 microns) for the electrons at the top (bottom) layer at ${V}_{g}$ = 2.5 V.

The transport mean free path ${l}_{tr}$ found to be is much longer than the effective quantum mean free path, *l*_*q*_ ≈ 100 nm, obtained from the decay of SdH oscillations. For sample TI1 we have found d ≈ 3.5(T) yielding *l*_*q*_ = 73 nm at ${n}_{t}$ = 1.2 10^15^ m^−2^. For sample TI5 k ≈ 2.8–3.2 (T) (see Fig. 4(b)), yielding *d* = *k*−*aT* ≈ 1.7–2.2 (T) at *T* = 4.2 K and a ≈ 0.28 T/K and, thus, *l*_*q*_ ≈ 110–140 (nm). The comparison of the two lengths suggests a substantial in-homogeneous broadening of SdH oscillations.
